# The influence of dexmedetomidine and propofol on circulating cytokine levels in healthy subjects

**DOI:** 10.1186/s12871-019-0895-3

**Published:** 2019-12-05

**Authors:** Minna Kallioinen, Annalotta Scheinin, Mikael Maksimow, Jaakko Långsjö, Kaike Kaisti, Riikka Takala, Tero Vahlberg, Katja Valli, Marko Salmi, Harry Scheinin, Anu Maksimow

**Affiliations:** 1 Department of Perioperative Services, Intensive Care and Pain Medicine, Turku University Hospital, POB 52, 20521 Turku, Finland; 2 Turku PET Centre, University of Turku and Turku University Hospital, Turku, Finland; 3 Medicity Research Laboratory, University of Turku, Turku, Finland; 4 Department of Intensive Care, Tampere University Hospital, Tampere, Finland; 5 Department of Anesthesiology and Intensive Care, Oulu University Hospital, Oulu, Finland; 6 Department of Clinical Medicine, Biostatistics, University of Turku and Turku University Hospital, Turku, Finland; 7 Department of Psychology and Speech-Language Pathology, and Turku Brain and Mind Centre, University of Turku, Turku, Finland; 8 Department of Cognitive Neuroscience and Philosophy, University of Skövde, Skövde, Sweden; 9 Institute of Biomedicine, University of Turku, Turku, Finland; 10 Integrative Physiology and Pharmacology, Institute of Biomedicine, University of Turku, Turku, Finland

**Keywords:** Dexmedetomidine, Propofol, Immunology, Immunosuppression, Cytokines

## Abstract

**Background:**

Surgery and diseases modify inflammatory responses and the immune system. Anesthetic agents also have effects on the human immune system but the responses they induce may be altered or masked by the surgical procedures or underlying illnesses. The aim of this study was to assess how single-drug dexmedetomidine and propofol anesthesia without any surgical intervention alter acute immunological biomarkers in healthy subjects.

**Methods:**

Thirty-five healthy, young male subjects were anesthetized using increasing concentrations of dexmedetomidine (*n* = 18) or propofol (*n* = 17) until loss of responsiveness (LOR) was detected. The treatment allocation was randomized. Multi-parametric immunoassays for the detection of 48 cytokines, chemokines and growth factors were used. Concentrations were determined at baseline and at the highest drug concentration for each subject.

**Results:**

The changes in the concentration of eotaxin (decrease after dexmedetomidine) and platelet-derived growth factor (PDGF, increase after propofol) were statistically significantly different between the groups. Significant changes were detected within both groups; the concentrations of monocyte chemotactic protein 1, chemokine ligand 27 and macrophage migration inhibitory factor were lower in both groups after the drug administration. Dexmedetomidine decreased the concentration of eotaxin, interleukin-18, interleukin-2Rα, stem cell factor, stem cell growth factor and vascular endothelial growth factor, and propofol decreased significantly the levels of hepatocyte growth factor, IFN-γ-induced protein 10 and monokine induced by IFN-γ, and increased the levels of interleukin-17, interleukin-5, interleukin-7 and PDGF.

**Conclusions:**

Dexmedetomidine seemed to have an immunosuppressive effect on the immune system whereas propofol seemed to induce mixed pro- and anti-inflammatory effects on the immune system. The choice of anesthetic agent could be relevant when treating patients with compromised immunological defense mechanisms.

**Trial registration:**

Before subject enrollment, the study was registered in the European Clinical Trials database (EudraCT number 2013–001496-21, The Neural Mechanisms of Anesthesia and Human Consciousness) and in ClinicalTrials.gov (Principal Investigator: Harry Scheinin, number NCT01889004, The Neural Mechanisms of Anesthesia and Human Consciousness, Part 2, on the 23rd of June 2013).

## Background

Surgery is known to trigger an inflammatory reaction [1], the magnitude of which depends on the type of surgery and the extent of tissue injury [2]. This postsurgical inflammatory reaction is followed by depression in cell mediated immunity, which in turn predisposes patients to postoperative infections and sepsis [3]. In addition, immunosuppression is also caused by volatile anesthetic agents [4, 5]. The effects of anesthetic agents on inflammatory cytokine profiles have previously been determined mostly in surgical patients or patients with critical illnesses, when the immunological status has inevitably been altered due to the surgical intervention, co-medication and/or the underlying disease [6–8]. Furthermore, since almost all of the studies on the effects of anesthetic drugs on immunological responses have been carried out in clinical settings, the patients have received various combinations and concentrations of other drugs such as opioids which also have effects on the humoral and cellular immunological responses [9]. Considering several confounding factors and wide methodological variation between the previous studies, it is not surprising that contradictory results have been obtained [4, 10].

Dexmedetomidine, a highly selective adrenergic α_2_-receptor agonist, is being used for short-term sedation for patients treated in the intensive care unit and also increasingly at the operating room during surgery. Propofol (2,6-diisopropylphenol) is a widely used intravenous anesthetic agent that acts via GABAergic transmitter system.

In vitro and in vivo experiments suggest that propofol impairs innate immune response, and thus could possess anti-inflammatory effects [6]. There is, however, contradictory evidence that propofol increases pro-inflammatory response in endotoxemia [7, 11]. Dexmedetomidine seems to have anti-inflammatory effects and be superior for sedating septic patients [12, 13]; yet, the effects of dexmedetomidine on cytokines, chemokines and growth factors have not been systematically studied.

In order to reveal direct anesthetic related effects on the immune system and compare immunological profiles of two anesthetic drugs, we administered either dexmedetomidine or propofol to healthy male subjects in a carefully standardized study setup.

## Methods

The samples for this immunological project were collected during a larger study “The Neural Mechanisms of Anesthesia and Human Consciousness (LOC-2013)” performed at the Turku University Hospital, Finland, after approval by the Ethics Committee of the Hospital District of Southwest Finland (Turku, Finland) and the Finnish Medicines Agency Fimea. Prior to enrolment of subjects, this study was registered in the European Clinical Trials database (EudraCT number 2013 001496 21) and in ClinicalTrials.gov (NCT01889004, Part 2, 23 Jun 2013). This manuscript adheres to the applicable CONSORT guidelines. More detailed description of the study has been published elsewhere [14].

### Study subjects

Thirty-five right-handed, healthy (American Society of Anesthesiologists physical status class I), non-smoking, 20–30-year-old male subjects participated in the study. The sample size differs from the original larger study (47 participants) due to technical and convenience issues in laboratory analysis and only 35 samples could be analyzed at the time. All participants underwent an interview and laboratory tests, including a hearing test, drug screen and an electrocardiogram. All subjects abstained from alcohol use or medication for 48 h prior to study sessions and fasted overnight. A written informed consent was acquired from all participants. Because the same subjects later underwent also positron emission tomography imaging and were exposed to radiation, only male subjects were considered eligible. The sample size was not based on a formal power calculation.

### Study treatments

Dexmedetomidine (Dexdor 100 μg/ml, Orion Pharma, Espoo, Finland) or propofol (Propofol-Lipuro 10 mg/ml, B. Braun, Melsungen, Germany) were administered intravenously via computer driven target-controlled infusions (TCI) aiming at pseudo steady-state plasma concentrations. A Harvard 22 syringe pump (Harvard Apparatus, South Natick, MA) connected to a portable computer running Stanpump software was used [15].

Subjects were randomized (permuted blocks) to receive either dexmedetomidine (*n* = 17) or propofol (*n* = 18) at escalating concentrations. For those receiving dexmedetomidine, the drug-infusion was started at target concentration of 1.0 ng/ml, followed first by 0.5 ng/ml target concentration increase and 0.25 ng/ml increments thereafter (i.e., 1.0–1.5 – 1.75 – 2.0 – 2.25 – etc. ng/ml) until loss of responsiveness (LOR) was achieved. The pharmacokinetic parameters by Talke et al. were applied [16]. For those receiving propofol, the drug-infusion was started at target concentration of 1.0 μg/ml, followed first by 0.5 μg/ml target concentration increase and 0.25 μg/ml increments thereafter (i.e., 1.0–1.5 – 1.75 – 2.0 – 2.25 – etc. μg/ml) until LOR was achieved. The pharmacokinetic parameters by Marsh et al. were applied [17]. After LOR the infusions were increased by 50% to reach a state assumed to represent the loss of consciousness, after which the drug infusions were terminated. For simplicity, we have used the term “anesthesia” to describe the achieved state even though no surgical stimulation was present.

Responsiveness was tested with a verbal stimulus scheme at each concentration level. The implementation of the session was guided by the responsiveness of the subject, as the emphasis in the study was on EEG-changes during the infusions. Therefore, the total duration of the infusions and the highest target dose varied between the subjects. All study sessions were held in the morning (drug administrations started at approximately 9 a.m.)

### Blood samples and immunological assays

Two forearm veins, one in each arm, were cannulated for administration of the anesthetic agents and for blood sampling. Ringer’s acetate infusion was used to keep the catheter open. Blood samples for the immunological assays were collected at baseline and at highest anesthetic concentration just before the drug infusion was terminated. Plasma concentrations of dexmedetomidine and propofol were determined using high-performance liquid chromatography as previously described [14].

Serum for the cytokine, chemokine and growth factor analyses was collected at baseline (without drug) and at highest anesthetic concentration (150% of the LOR concentration). From each venous blood sample drawn for drug plasma concentration measurements, a 100 μl aliquot of serum was frozen separately at − 70 °C until analyses. The changes in the immunological signaling molecules between the baseline and the highest concentration were determined for each subject. All analyses were performed in a single assay run using the Bio-Plex Pro Human Cytokine 21- and 27-plex magnetic bead suspension array kits (Bio-Rad Laboratories, Hercules, CA, USA) as described previously [18]. Results were analyzed using the Bio-Plex 200 System and Bio-Plex Manager 6.0 software (Bio-Rad Laboratories). The 21-plex panel contained interleukin 1α (IL-1α), IL-2 receptor α (IL-2Rα), IL-3, IL-12p40, IL-16, IL-18, cutaneous T cell-attracting chemokine (CTACK), growth-regulated oncogene α (GROα), hepatocyte growth factor (HGF), interferon α2 (IFN-α2), leukemia inhibitory factor (LIF), monocyte chemotactic protein 3 (MCP-3), macrophage colony-stimulating factor (M-CSF), macrophage migration inhibitory factor (MIF), monokine induced by IFN-γ (MIG), β-nerve growth factor (β-NGF), stem cell factor (SCF), stem cell growth factor-β (SCGF-β), stromal cell-derived factor 1α (SDF-1α), tumor necrosis factor β (TNF-β), and TNF-related apoptosis inducing ligand (TRAIL) assays. The 27-plex contained IL-1β, IL-1 receptor antagonist (IL-1ra), IL-2, IL-4, IL-5, IL-6, IL-7, IL-8, IL-9, IL-10, IL-12p70, IL-13, IL-15, IL-17, basic fibroblast growth factor (bFGF), eotaxin, granulocyte colony-stimulating factor (G-CSF), granulocyte-macrophage colony-stimulating factor (GM-CSF), IFN-γ, IFN-γ-induced protein 10 (IP-10), monocyte chemotactic protein 1 (MCP-1), macrophage inflammatory protein 1α (MIP-1α), MIP-1β, platelet-derived growth factor (PDGF), regulated on activation normal T cell expressed and secreted (RANTES), TNF-α, and vascular endothelial growth factor (VEGF) assays. The persons who conducted the cytokine measurements were unaware of the status and anesthetic drug of the subjects.

### Statistical analysis

Data were analyzed using nonparametric methods due to the skewed distributions and the outlying observations. Mann-Whitney U-test was used to test the difference in the change in the concentrations between the groups. In addition, the changes within drug groups were tested with Wilcoxon signed rank test.

In multiplex assays, intra-assay variation (IAV) varies between assays and needs to be defined for each analyte. The mean IAV in our study was 6.8%. Therefore, a cut-off point was set to 10%, i.e., only concentration changes of at least 10% between the median baseline and the highest anaesthetic concentration were considered relevant and are reported for the within drug analyses. 10% cut-off is commonly used in reporting multiplex assay results.

To adjust for multiple testing, Benjamini-Hochberg procedure was applied to control the false discovery rate at 0.05 [19]. Statistical analyses were performed using SAS System for Windows, version 9.4 (SAS Institute Inc., Cary, NC). *P*-values lower than 0.05 were considered as statistically significant.

## Results

The administration of the anesthetic was performed successfully in all 35 subjects. No adverse events or clinically significant changes in the vital parameters were observed in any of the study participants (data not shown). The highest mean (standard deviation, (SD)) measured drug concentration was 3.19 (0.89) ng/ml for dexmedetomidine and 2.65 (0.78) μg/ml for propofol. The average infusion time was 125 (26) min for dexmedetomidine (range 79–166 min) and 100 (30) min for propofol (range 49–153 min).

There were no significant differences in the concentrations of the immunological analytes between the groups at baseline. In the 21-plex panel, ten of the analytes were below the detection limit and in the 27-plex panel, three of the analytes were below the detection limit and one was above.

**Fig. 1:**
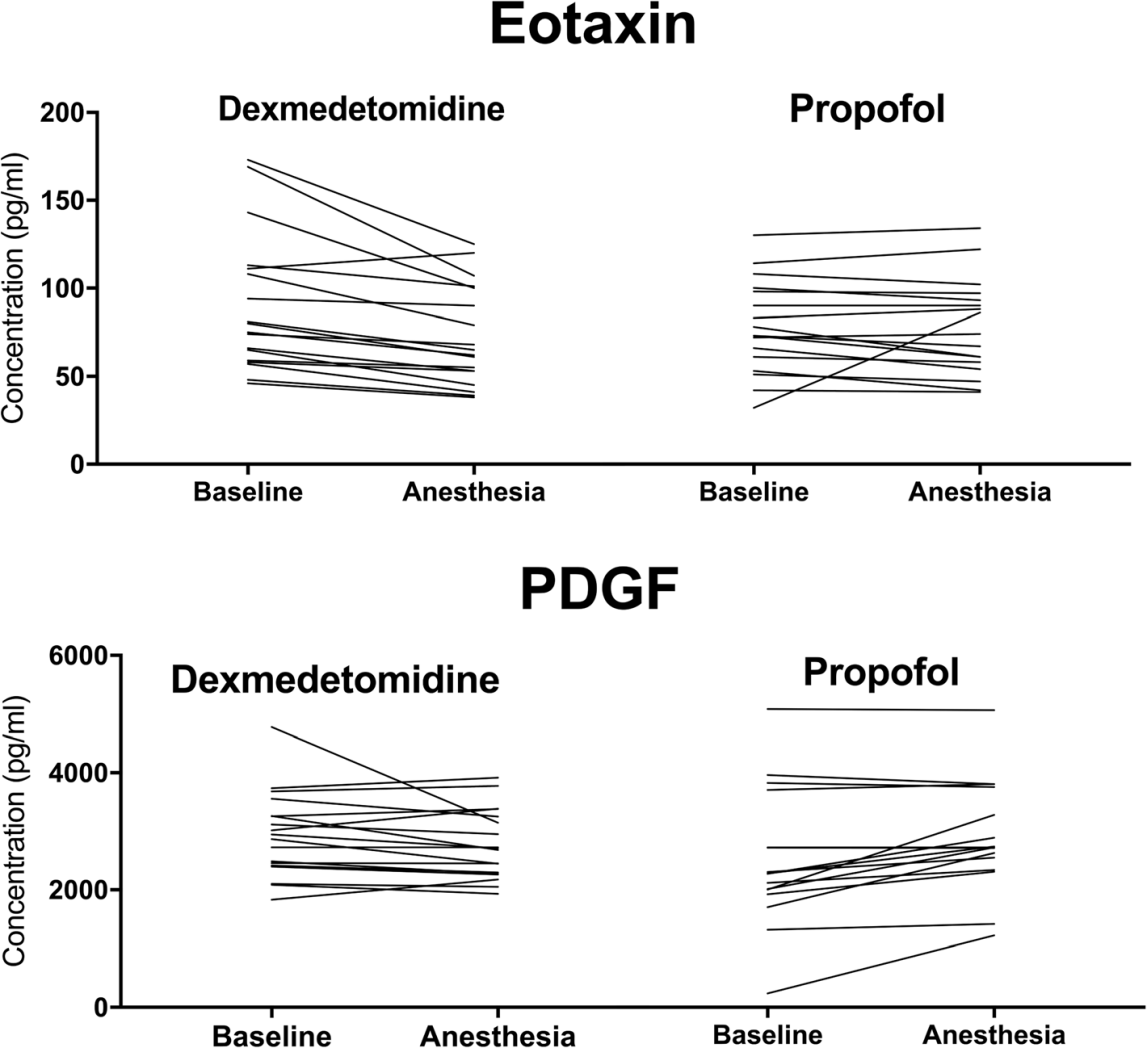
Individual Eotaxin and PDGF concentrations at baseline and during dexmedetomidine and propofol anesthesia. The changes were statistically significantly different between the groups (*p* = 0.036 and *p* = 0.022, respectively; Mann-Whitney U-test corrected for multiple comparison)

The changes in the concentration of eotaxin and PDGF were significantly different between the groups (for eotaxin *p* = 0.036 and for PDGF *p* = 0.022, respectively; Mann-Whitney U-test corrected for multiple testing). In the dexmedetomidine group, eotaxin decreased after drug administration whereas in the propofol group PDGF increased (Fig. 1).

There were statistically significant ≥10% changes in 9 analytes in the dexmedetomidine and 10 analytes in the propofol group. Within the groups, both dexmedetomidine and propofol decreased significantly the levels of MCP-1, CTACK and MIF. In addition, dexmedetomidine decreased significantly the level of eotaxin, IL-18, IL-1rα, SCF, SCGF and VEGF. Propofol decreased significantly the concentrations of HGF, MIG and IP-10, and increased the concentrations of IL-5, IL-7, IL-17 and PDGF (Tables 1 and 2).

**Table 1:** Statistically significant ≥10% decreases of 9 cytokines in the dexmedetomidine group

Cytokine	Baseline pg/ml [median (IQR)]	Anesthesia pg/ml [(median (IQR)]	Unadjusted p-value	Adjusted p-value
MCP-1	40 (16)	30 (12)	< 0.001	< 0.001
CTACK	776 (171)	608 (227)	< 0.001	0.002
MIF	180 (160)	123 (160)	< 0.001	< 0.001
Eotaxin	77 (52)	64 (48)	< 0.001	< 0.001
SCF	64 (15)	56 (16)	< 0.001	< 0.001
IL-18	44 (22)	36 (15)	0.012	0.038
IL-2rα	42 (17)	34 (12)	0.006	0.018
VEGF	54 (62)	47 (42)	0.016	0.04
SCGF	16,305 (4218)	13,006 (4003)	< 0.001	< 0.001

Data were analyzed using nonparametric testing due to the skewed distributions and the outlying observations. The median as well as the inter quartile range (IQR) values are reported for each cytokine. The unadjusted *p*-values were calculated using Wilcoxon signed rank test and the adjusted *p*-values were calculated using Benjamini-Hochberg method (42 hypothesis tests) controlling for a false discovery rate at 0.05

**Table 2:** Statistically significant ≥10% changes of 10 cytokines in the propofol group

Cytokine	Baseline pg/ml [median (IQR)]	Anesthesia pg/ml [median (IQR)]	Unadjusted p-value	Adjusted p-value
PDGF	2160 (460)	2625 (971)	0.002	0.02
IP-10	332 (188)	289 (112)	0.004	0.03
IL-5	6 (1)	8 (2)	0.006	0.03
IL-7	10 (4)	11 (3)	0.005	0.03
IL-17	158 (60)	175 (71)	0.008	0.03
MIG	245 (81)	201 (69)	< 0.001	0.01
HGF	156 (24)	129 (33)	0.002	0.02
MCP-1	38 (16)	31 (14)	0.004	0.03
CTACK	598 (270)	534 (168)	0.005	0.03
MIF	227 (159)	113 (82)	< 0.001	0.01

Data were analyzed using nonparametric testing due to the skewed distributions and the outlying observations. The median as well as the inter quartile range (IQR) values are reported for each cytokine. The unadjusted p-values were calculated using Wilcoxon signed rank test and the adjusted p-values were calculated using Benjamini-Hochberg method (42 hypothesis tests) controlling for a false discovery rate at 0.05

We also investigated the effects of dexmedetomidine and propofol to the ratio of Th1 to Th2 cytokines, specifically IFN-γ to IL-4 and IL-5, but we found no statistically significant differences.

## Discussion

In the present study, we collected samples from healthy subjects anesthetized with either dexmedetomidine or propofol and assessed drug-induced changes in 48 immunological analytes. The samples were collected during a larger study investigating the neural mechanisms of anesthesia and human consciousness [14]. Our study is unique in three ways: we evaluated cytokine, chemokine and growth factor profiles in *healthy subjects* receiving *single-agent anesthesia* of either dexmedetomidine or propofol *without any surgical intervention or other nociceptive stimuli*. Within the groups we observed significant changes in the concentrations of numerous inflammatory chemokines and cytokines, and between the groups we observed a significant difference in the concentrations of PDGF and eotaxin.

Within the groups, we found that dexmedetomidine and propofol both affect the levels of several inflammatory chemokines. Chemokines are chemoattractive cytokines that primarily regulate the migration of peripheral immune cells [20]. In our study, both dexmedetomidine and propofol significantly decreased the concentrations of MCP-1, CTACK and MIF.

We observed that dexmedetomidine decreased the concentrations of IL-18, IL-2Rα, SCF, SCGF and VEGF. Interestingly, we found that only dexmedetomidine significantly decreased the concentration of eotaxin. Eotaxin is a potent eosinophil chemoattractant that mediates leukocyte recruitment in allergic diseases such as asthma [21]. Nevertheless, eotaxin is also broadly expressed in tissues void of eosinophils and is strongly up-regulated in murine model of sepsis [22], which suggests that eotaxin may have formerly unknown functions. Previously, eotaxin has been identified as an important factor responsible for aging-associated weakening in hippocampal neurogenesis as well as in learning and memory [23]. This finding may be important considering that dexmedetomidine has been shown to reduce the incidence of post-operative cognitive dysfunction [24].

Earlier studies on surgical patients have demonstrated that dexmedetomidine decreases postoperative levels of pro-inflammatory cytokines [25, 26], which are in accordance with our results. In addition, dexmedetomidine has been shown to have notable anti-inflammatory properties [27], also when compared to propofol [11]. In animal studies dexmedetomidine has been shown to attenuate the immune response and to improve survival in experimental sepsis [28–30]. These properties are most likely due to dexmedetomidine’s sympatholytic activity as demonstrated by Hofer et al. [30] and Xiang et al. [28].

We found that propofol significantly increased the levels of IL-5, IL-7, IL-17 and PDGF. These pro-inflammatory interleukins play a substantial role in adaptive immune response. However, propofol also decreased the levels of the anti-inflammatory HGF, IP-10 and MIG. Thus, the findings for propofol are somewhat contradictory. The increase in the concentration of many pro-inflammatory cytokines may suggest that propofol could have a pro-inflammatory effect on the immune system mainly by increasing the activation of lymphocytes and, thus, adaptive immunity. Nevertheless, propofol also seems to have a slight suppressive effect on innate immunity by decreasing the levels of several pro-inflammatory chemokines. The decreases in the levels of HGF, IP-10 and MIG are particularly interesting. The overexpression of HGF has been associated with a number of different cancers [31]. Furthermore, IP-10 and MIG are also linked to tumor development and are being investigated as possible treatment targets in cancer research [32, 33].

There is evidence that propofol could be superior to volatile anesthesia in cancer patients [34–37]. In an extensive retrospective analysis, an increased hazard ratio for death was observed in patients receiving volatile anesthesia (versus propofol) [35]. The anti-inflammatory properties of volatile anesthetics is well established [37] and is shown to accelerate the growth of neoplastic cells and enhance metastasis [5]. Therefore, patients suffering from cancer could benefit from the individual choice of a certain anesthetic agent [34, 38]. The mixed pro and anti-inflammatory response that propofol seems to induce could be beneficial for cancer patients. However, the evidence is greatly controversial at the moment [39] [40] and prospective randomized controlled studies are needed to establish the influence of different anesthetics on oncological outcomes. In studies with rodents, dexmedetomidine has been shown to promote metastasis in breast, lung and colon cancer [41]. Respectively, in a recent retrospective clinical study the intraoperative use of dexmedetomidine was associated with decreased overall survival after lung cancer surgery [42]. This unfavorable effect could be due to direct stimulation of cancer cells by dexmedetomidine, or the induction of immunosuppression. Similarly, prospective clinical studies are needed to confirm these findings.

An intact and healthy immune system is by default essential in fighting against illness and infection. An acute episode of sepsis is characterized by an extensive release of cytokines and other mediators resulting in a dysregulated immune response leading to organ injury or even death. In theory, attenuation of this immune response would perhaps be beneficial in the early stages of sepsis to avoid organ damage and adverse outcome. In septic patients, propofol could be unfavorable since it may worsen the endotoxemia by increasing the levels of pro-inflammatory cytokines [7, 43]. Interestingly, in septic patients the use of dexmedetomidine has been associated with lower proinflammatory response and improved outcome compared to propofol [11, 12, 27]. However, the findings are conflicting and in a very recently published study by Shehabi et al. studying the effects of early sedation with dexmedetomidine in critically ill patients, there was no difference in 90-day mortality between dexmedetomidine and usual-care [44]. In addition, there were more adverse events in the dexmedetomidine group and, according to the subgroup analysis, the results were equal for septic patients.

We also investigated the effects of dexmedetomidine and propofol to the Th1/Th2 balance by measuring the changes in the ratio of a Th1 cytokine, IFN-γ, to Th2 cytokines, IL-4 and IL-5. The Th1/Th2 balance could indicate the status and readiness of the immune system to react against pathogens. However, we did not find significant changes in the Th1/Th2 ratio with either drug. It is intriguing that even though propofol increases the levels of several pro-inflammatory cytokines, it does not shift the Th1/Th2 balance significantly to either side.

The strength of our study is that the healthy subjects were anesthetized using only one anesthetic agent without any surgical intervention or nociceptive stimuli, resulting in native drug induced cytokine response. In clinical situations where the immune system is already modified by surgery or the underlying disease, such as sepsis or cancer, it is not possible to determine the immunological effects prompted solely by the anesthetic agent. Furthermore, in contrast to most of the previous studies that included only 6–8 immunological analytes [8, 45], our multi-parametric assay consisting of 48 analytes offers a broader spectrum in characterizing the anesthetic-induced alterations in acute immune response caused by two different, commonly used anesthetic drugs. However, contrary to most previous studies, the samples in our study were taken immediately after the exposure to the drug and there was no follow-up period. This means that the observed changes are only immediate reactions and that we might have seen more pronounced or somewhat different changes in the concentrations had we taken samples 1–3 days after the exposure. In spite of the observed statistically significant changes in the levels of the reported analytes, it can be discussed due to a short observation period that responses requiring de novo synthesis may not have been detected, whereas acute responses resulting from release from storage vesicles were revealed. It must also be taken into account that the immunological response these anesthetics induce may and will likely differ under stress such as surgery or acute illness. Therefore, the results obtained in healthy volunteers cannot be directly applied to the treatment of critically ill patients.

One important limitation in our study is that we did not have a control group without anesthesia. Therefore, the possible impact of circadian rhythm of the measured cytokines and mediators remains ambiguous. Another limitation is that we only included young males in our study, and therefore the impact of gender or age on the results is not known.

## Conclusions

The present study investigated the immediate drug-induced changes in a large array of immunological analytes in healthy males receiving either dexmedetomidine or propofol. Both drugs affected the levels of several cytokines and growth factors. Dexmedetomidine seems to have a distinctly immunosuppressive effect and propofol a partly pro-inflammatory but also slightly anti-inflammatory effect on the immune system. The possible clinical implications of these results warrant controlled studies in different patient populations.

## Data Availability

The datasets used and/or analyzed during the current study are available from the corresponding author on reasonable request.

## Abbreviations

(IFN-γ) IL: Interleukin e.g. interleukin-6 (IL-6)
CD: Cluster of differentiation
CLA: Cutaneous lymphocyte-associated antigen
CTACK: Cutaneous T cell-attracting chemokine
GABA: Gamma-aminobutyric acid
HGF: Hepatocyte growth factor
ICU: Intensive care unit
IFN: Interferon e.g. interferon-γ
IP-10: Interferon gamma-induced protein 10
IQR: Interquartile range
LOR: Loss of responsiveness
MCP: Monocyte chemotactic protein e.g. monocyte chemotactic protein 1 (MCP-1)
MIF: Macrophage migration inhibitory factor
MIG: Monokine induced by gamma interferon
PDGF: Platelet-derived growth factor
SCF: Stem cell factor
SD: Standard deviation
STGF: Stem cell growth factor
Th: T helper e.g. Type 1 T helper (Th1)
TIVA: Total intravenous anesthesia
TNF: Tumor necrosis factor e.g. tumor necrosis factor α (TNF-α)
VEGF: Vascular endothelial growth factor
